# Nitrogen defect-containing polymeric carbon nitride for efficient photocatalytic H_2_ evolution and RhB degradation under visible light irradiation†

**DOI:** 10.1039/d2ra04928g

**Published:** 2022-08-31

**Authors:** Man Li, Xin Bai, Xi Rao, Shaohui Zheng, Yongping Zhang

**Affiliations:** School of Materials and Energy, Southwest University Chongqing 400715 China shaohuizheng@swu.edu.cn zhangyyping6@swu.edu.cn

## Abstract

Introducing defects in polymeric carbon nitride (CN) in a predetermined way is a great challenge to explicate the effect of defects on the photocatalytic activity. Herein, we provide a pathway to synthesize g-C_3_N_4_ with nitrogen defects by simply calcining melamine and trithiocyanuric acid at elevated temperature. Nitrogen defects at the N-bridging sites lead to an intermediate energy gap between the valence band and the conduction band, which greatly increases the photon absorption in the visible light range. Electron paramagnetic resonance (EPR) and photoluminescence (PL) verify that the significantly improved light utilization efficiency and rapid charge transfer correlate with nitrogen defects. The hydrogen evolution rate of 2SCN reached 41.4 μmol h^−1^, about 20.7 times that of pure g-C_3_N_4_, and its degradation rate for rhodamine B (RhB) is about 2.5 times that of CN. The experimental results proved that the photoinduced electron–hole pairs react with adsorbed O_2_ to form ˙O_2_^−^, facilitating the photodegradation of organic pollutants.

## Abstract 2

Nitrogen defects formed by missing the inner N-bridge atoms and sulfur dopant in g-C_3_N_4_.

## Graphical abstract

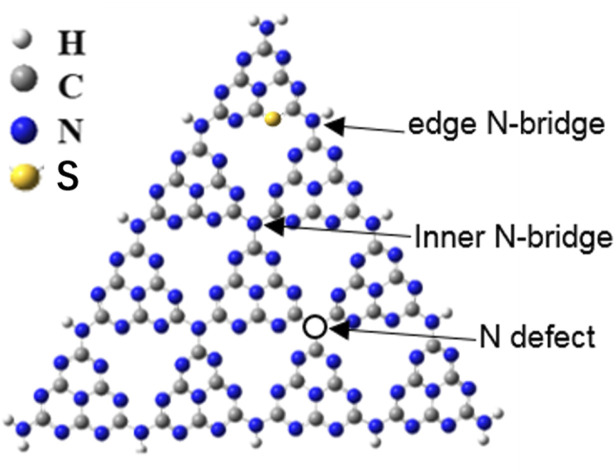

## 1. Introduction

Polymeric carbon nitride (g-C_3_N_4_), a direct band gap semiconductor with a two-dimensional graphene like structure, is considered to be one of the most promising materials in photocatalytic pollutant degradation and photocatalytic hydrogen evolution *via* water splitting due to its high stability, low cost, non-toxicity, environmental friendliness and appropriate band gap.^1–3^ However, some shortcomings, such as the small specific surface area, high recombination rate of photogenerated electrons/holes and low absorption efficiency of visible light, restricted the pristine polymeric carbon nitride in photocatalytic application. Many perspectives were explored to improve its photocatalytic activity, including element doping,^4–6^ forming semiconductor heterojunctions,^7^ engineering defects in catalysts,^8^ and metal cluster loading.^9^

It has been proved that the construction of defects in g-C_3_N_4_ can promote the transmission of charge carriers, so as to effectively enhance the photocatalytic activity. According to previous studies, it is found that higher specific surface area can be obtained after strong acid treatment, which increases the number of active sites.^10,11^ Recently, many workers found that polymeric carbon nitride with defective structure can be obtained by treating the precursor of polymeric carbon nitride with the weak reducibility of organic acid with weak acidity.^12^ It can not only increase the specific surface area, but also produce defects, which is conducive to the utilization of charge carriers.^13^ According to different preparation methods, carbon defects or nitrogen defects can be obtained,^14,15^ which will also form a sub band gap, so as to greatly expand the optical response and improve the survival elapse of photogenerated carriers. Moreover, defects can induce greater specific surface area and pore structure, increase light absorption area and improve light absorption capacity,^16,17^ which are conducive to photocatalytic reaction. At molecular scale, the defect origin and site were not well established in those studies. Further experimental and theoretical explorations were needed to understand defect structure and its effect on photocatalytic activity.

**Scheme 1:**
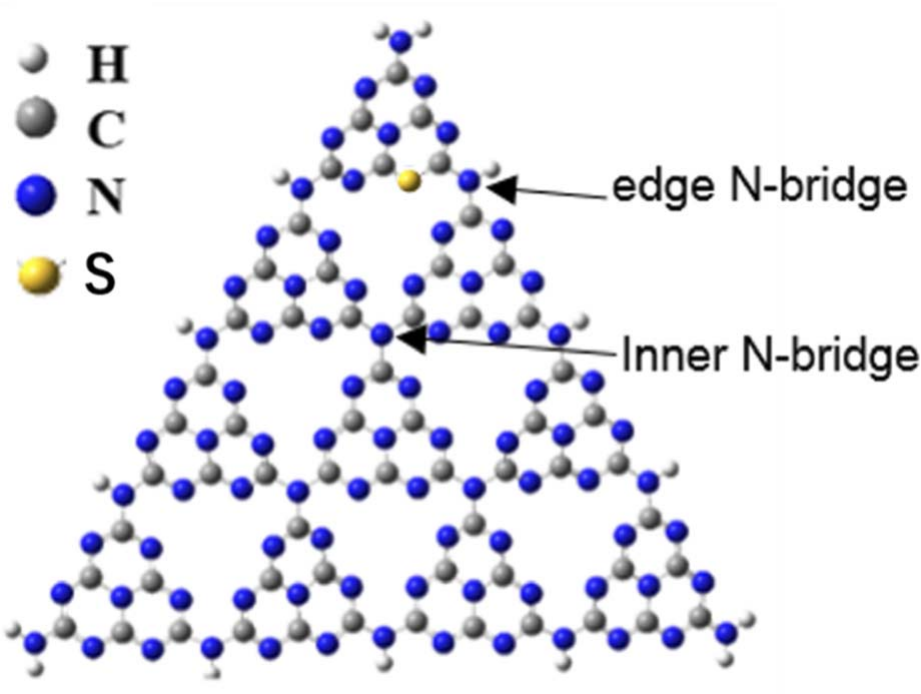
Schematic illustration of g-C_3_N_4_ structure.

The crystallinity, degree of polymerization, defect formation of C_3_N_4_ molecular structure exhibit profound effect on its photocatalytic performance. Our recent study revealed that nitrogen defects in polymeric carbon nitride molecules by cutting the network nodes is the main factor to enhance the photocatalytic performance, besides the crystallinity and polymerization degree.^18^ Some literature reported that nanostructured and S-doping g-C_3_N_4_ catalysts were prepared with similar co-polymerization method by calcining melamine and trithiocyanuric acid, and ascribed the enhanced photocatalytic performance to synergetic effect of extended light absorption and more catalytic sites.^19–21^ However, the detailed molecular structure of g-C_3_N_4_ remained unexplored. How to isolate the solitary defect factor and study its effect on photocatalytic activity is especially important for understanding the mechanism of defects. There exist two kind of sp^3^ tertiary N–[C]_3_ nitrogen vacancies, inner N-bridge vacancies characterized the N defects and edge N-bridge vacancies increased the amino group C–NH_*x*_, as shown in Scheme 1. We proposed a pathway to introduce nitrogen vacancy in the sp^3^ N–[C]_3_ linkage by calcining melamine and trithiocyanuric acid, in order to clarify the effect of nitrogen defect on the photocatalytic enhancement.

In this work, a series of g-C_3_N_4_ with nitrogen defects and pore structure were prepared by simple thermal condensation of melamine and trithiocyanuric acid. Due to the increase of reaction active sites and abundant defect structures, the photocatalytic activity of the samples has been greatly improved.

## 2. Experimental details

### 2.1 Reagents

Melamine (C_3_H_6_N_6_, 99%), and trithiocyanuric acid (C_3_H_3_N_3_S_3_, 95%) were purchased from Sinopharm Chemical Reagent Co. Ltd. All chemicals were analytical grade and used without further purification. Deionized water was used throughout this study.

### 2.2 Catalyst preparation

0.01 mol melamine and different amount of trithiocyanuric acid (0.0075 mol, 0.01 mol, 0.0125 mol) were dissolved in 60 ml deionized water under ultrasonic stirring for 12 h, then dried in an oven at 60 °C for 3 h. The dried precipitates were heated at 600 °C for 2 h in a tubular furnace at N_2_ environment, with a ramp rate of 3 °C min^−1^, respectively. Accordingly, the catalysts were denoted as 1SCN, 2SCN and 3SCN for trithiocyanuric acid of 0.0075 mol, 0.01 mol, and 0.0125 mol, respectively. 0.01 mol melamine was heated at 600 °C for 2 h through a similar process without adding trithiocyanuric acid to obtain the pure g-C_3_N_4_, denoted as CN.

### 2.3 Characterization

Morphologies and structures were observed using a thermal field emission scanning electron microscope (FESEM, JSM-7800F), transmission electron microscope (TEM, Zeiss Libra 200FE), and X-ray diffraction (XRD, Shimadzu XRD7000) with Cu Kα as radiation source (*λ* = 1.5418 Å). The vibration state of chemical states were measured by Fourier transform infrared spectroscopy (FTIR, Model Frontier), X-ray photoelectron spectroscopy (XPS, VG ESCALAB 250Xi) with radiation source of Al Kα (*hν* = 1486.8 eV). The UV-vis diffuse reflectance spectra were performed on an Agilent Cary 5000 UV-vis NIR system with 100% BaSO_4_ as the reflection sample. The photoluminescence (PL) spectra were recorded on a Hitachi F-7000 spectrophotometer with a 150 W xenon light as the excitation source. N_2_ adsorption–desorption isotherms were carried out on a Quadrasorbevo 2QDS-MP-30 specific surface area tester (BET, Quadrasorbevo 2QDS-MP-30). The transient photocurrent response curve (*I*–*t*), Mott–Schottky curve, electrochemical impedance spectroscopy (EIS) were performed using AUTOLAB (model PGSTAT302N) electrochemical workstation. A 500 W xenon lamp was used as the light source, and 0.25 M Na_2_SO_4_ solution as the electrolyte. Free radical trapping experiments were carried out on electron paramagnetic resonance (EPR) spectrometer (Bruke, EMXnano) with 420 nm LED as excitation source.

### 2.4 Photocatalytic hydrogen evolution

The photocatalytic hydrogen evolution evaluation was carried out in a photocatalysis evaluation system (Suncat Instrument, Beijing). The 500 W Xe lamp (zolix, gloria-x500a) with intensity of 110 mW cm^−2^ was used as the simulated solar light with a wavelength *λ* ≥ 420 nm. The reactor was maintained at 20 °C with an external circulation cooling system. 10 mg photocatalyst was dispersed in 30 ml aqueous solution with 17 vol% triethanolamine (TEOA) as sacrificing agent and 3 wt% Pt ion (H_2_PtCl_6_ H_2_O) as co-catalyst. Before turning on the light, the reactor is pumped to a high vacuum of 10^−8^ torr, and then filled with argon. Under the light irradiation process, the reaction suspension is stirred continuously. 1 ml of gas was extracted automatically from the reactor at interval of 30 min, and analyzed with a gas chromatograph (GC-2018, Shimadzu) with TDX-01 molecular sieve, thermal conductivity detector and Ar carrier gas.

### 2.5 Photocatalytic removal of rhodamine B (RhB)

10 mg photocatalyst was dispersed in 60 ml rhodamine B aqueous solution (25 mg L^−1^). The light source was a 300 W HSX-F300 xenon lamp. The solution was kept in the dark for 30 min to reach the adsorption equilibrium. Then the solution was exposed to visible light for degradation. At 3 min interval, 1 ml suspension was taken out and centrifuged, and measured the characteristic UV-vis absorption spectra. The maximum absorption peak was recorded and used to evaluate the concentration of RhB. The degradation rate of RhB aqueous solution at time *t* can be calculated by the following formula:Degradation rate = (1 − *C*_*t*_/*C*_0_) × 100%where *C*_0_ is the adsorption–desorption equilibrium concentration of RhB, and *C*_*t*_ is the RhB concentration at irradiation time *t*.

## 3. Results and discussion

**Fig. 1:**
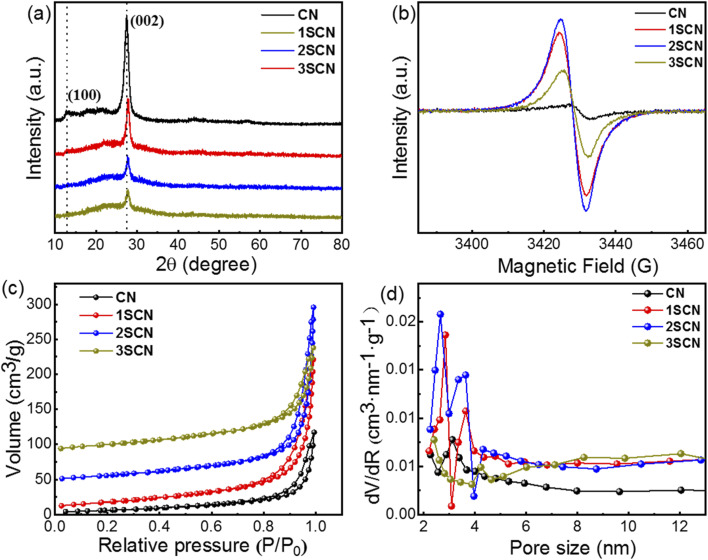
XRD patterns (a), EPR spectra (b) BET isotherms (c) and pore size distribution (d) of CN and SCN at different nitrogen defects.

Catalysts 1SCN, 2SCN, and 3SCN presents a similar XRD patterns as CN, shown in Fig. 1 (a), in which the strong peak at 27.3° is attributed to the (002) diffraction plane of the interplanar stacking of conjugated aromatic units, and the weak (100) at 13.1° is associated with the in-plane repeated motif of the tri-*s*-triazine ring.^22–25^ Detailed analyses revealed the intensity of the (002) diffraction peaks for SCN decreases dramatically compared to CN with introducing nitrogen defects and S doped g-C_3_N_4_, which indicates that introducing certain amount of nitrogen defects reduces the compactness of repeating layers. EPR signals in Fig. 1(b) showed the obvious symmetrical peak with *g* = 2.0038, characterizing the density of nitrogen defects. The weak signal for pure CN is caused by the unpaired C atom in the aromatic heterocycles in g-C_3_N_4_.^26^ As for SCN, the signal for unpaired electronic augments drastically, indicating the increase nitrogen defects. With the increase of ratio of trithiocyanuric acid, the nitrogen defect content increases accordingly, then reaches a plateau at certain point, further increase the defects means more missing the edge N-bridge atoms, thus explains that 2SCN has highest defects density. N_2_ adsorption–desorption isotherms in Fig. 1(c) depicted that all catalysts exhibit a typical type IV isotherm with a H3 hysteresis loop, indicating the presence of mesopores.^27^ The specific surface area is 23.2 m^2^ g^−1^, 65.3 m^2^ g^−1^, 70.8 m^2^ g^−1^, and 54.6 m^2^ g^−1^, for CN, 1SCN, 2SCN, and 3SCN, respectively. That indicates that the introducing nitrogen defects have a significant effect on the specific surface area of the samples, and 2SCN with highest specific surface area among all samples. The total pore volume is 0.16 m^3^ g^−1^, 0.32 m^3^ g^−1^, 0.38 m^3^ g^−1^, and 0.22 m^3^ g^−1^, for CN, 1SCN, 2SCN, and 3SCN, respectively. The corresponding mesoporous distribution of the sample is shown in Fig. 1(d). The mesoporous size is mainly distributed in the range of 2–6 nm. The mesoporous distribution on the material surface helps to increase the specific surface area of the material and enhance the photocatalytic activity. The presence of mesopores favors multiple light scattering/reflection, resulting in enhanced harvesting of the exciting light and thus improved photocatalytic activity.^28^

**Fig. 2:**
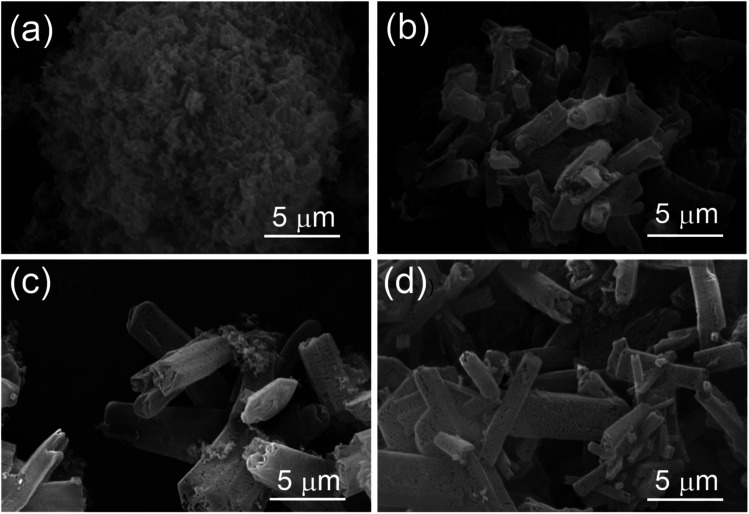
SEM images of CN (a), 1SCN (b), 2SCN (c), and 3SCN (d).

SEM images in Fig. 2(a) showed that the pure g-C_3_N_4_ appeared as the stacked bulk structures. SCN appeared as irregular nanorods with length of around 5 μm and diameter around several hundred micrometers. There existed many microporous features and small cracks, the density of micro-cracks and pores on the surface of the samples increase, as shown in Fig. 2(b–d). That is consistent with the BET data.

**Fig. 3:**
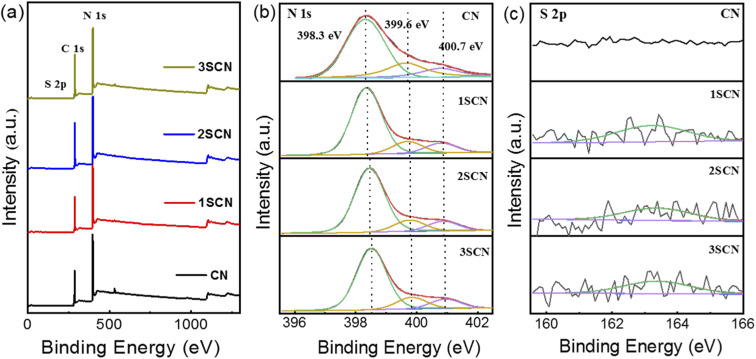
XPS core level survey spectra (a), and high-resolution N 1s (b), and S 2p (c) of the samples.

XPS survey spectra in Fig. 3(a) showed there exist the C 1s, and N 1s signals, and the signal for S is almost illegible. XPS N 1s spectra in Fig. 3(b) could be fitted into three peaks with binding energy at 398.5 eV, 399.7 eV, 400.8 eV, corresponding to sp^2^ aromatic N atoms in aromatic tri-*s*-triazine C–N skeleton (N1, C–N=C), sp^3^ hybrid tertiary amine N atoms (N2, N–[C]_3_), amino functional groups at the edge of the aromatic ring plane (N3, C–NH_*x*_).^29–32^ XPS S 2p spectra in Fig. 3(c) showed the S atoms mainly exist in one state with binding energy of 163.1 eV, corresponding to the C–S bond formed by substituting N atom in the aromatic tri-*s*-triazine. The S atomic ratio remains relatively low for samples 1SCN, 2SCN, and 3SCN, indicating only fractional S atoms were doped in g-C_3_N_4_. There exist two kind of sp^3^ tertiary N–[C]_3_ nitrogen vacancies, inner N-bridge vacancies characterized the N defects and edge N-bridge vacancies increased the amino group C–NH_*x*_. The percentage of N-containing species is listed in Table 1. With the increase of trithiocyanuric acid, the percentage of N atoms in amino groups (N_3_, C–NH_*x*_) increases gradually, and the ratio of N–[C]_3_ nitrogen decreases accordingly. The percentage of the bridging tertiary N atoms (N2, N–[C]_3_) decrease, indicating some tertiary N atoms are missing to form nitrogen defects located at the tertiary nitrogen lattice sites.^18^ The inner N-bridge vacancies are related to the N defects, and formation of edge N-bridge vacancies induces the additional NH_*x*_ group. That explains why 2SCN has highest content of N defects observed in EPR spectra.

**Table 1:** The component ratios of the N 1s spectra for different samples

Samples	Binding energy (eV)	Peak assignment	Atomic percentage
CN	398.3	C–N=C (sp^2^)	76.14
	399.4	N–[C]_3_ (sp^3^)	13.43
	400.7	C–NH_X_	10.43
1SCN	398.4	C–N=C (sp^2^)	76.18
	399.7	N–[C]_3_ (sp^3^)	12.97
	400.8	C–NH_*X*_	10.85
2SCN	398.5	C–N=C (sp^2^)	76.11
	399.7	N–[C]_3_ (sp^3^)	12.86
	400.8	C–NH_*X*_	11.03
3SCN	398.5	C–N=C (sp^2^)	76.14
	399.8	N–[C]_3_ (sp^3^)	12.77
	400.9	C–NH_*X*_	11.09

**Fig. 4:**
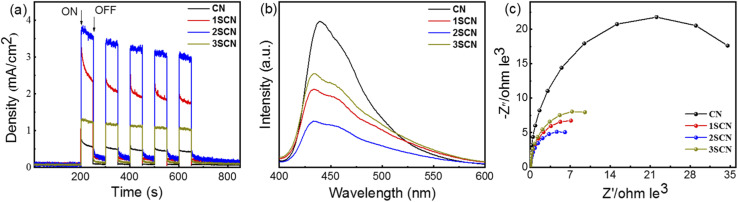
Transient photocurrent responses (a), PL spectra (b), and EIS (c) of the samples.

The transient photocurrent response curve in Fig. 4(a) shows that the photocurrent response of S-doped g-C_3_N_4_ increases according the trend of nitrogen defect density.^33–35^ All samples can respond continuously and stabilizedly under the same continuous bias voltage, with 2SCN exhibits the highest photocurrent responses. Fig. 4(b) shows the PL spectrum of the sample at the excitation wavelength of 373 nm. Obviously, the pure phase CN has a strong emission peak near 442 nm. The PL intensity of SCN samples is much lower than that of the pure phase CN. With the increase of N defects, the PL intensity of the sample becomes weaker, and the fluorescence quenching of 2SCN is the most obvious.^36,37^ EIS in Fig. 4(c) showed that the order of arc radius is 2SCN < 3SCN < 1SCN < CN, which proves that the migration rate of surface migration rate of SCN samples becomes faster with the increase of defect content. The results show that the separation efficiency of photogenerated carriers of the sample has been significantly improved.^35^ The above results confirm that the introduction of nitrogen defects enhances the electron hole separation efficiency, reduces the charge transfer resistance, improves the stability of charge carriers and enhances the photocurrent density.^38^

**Fig. 5:**
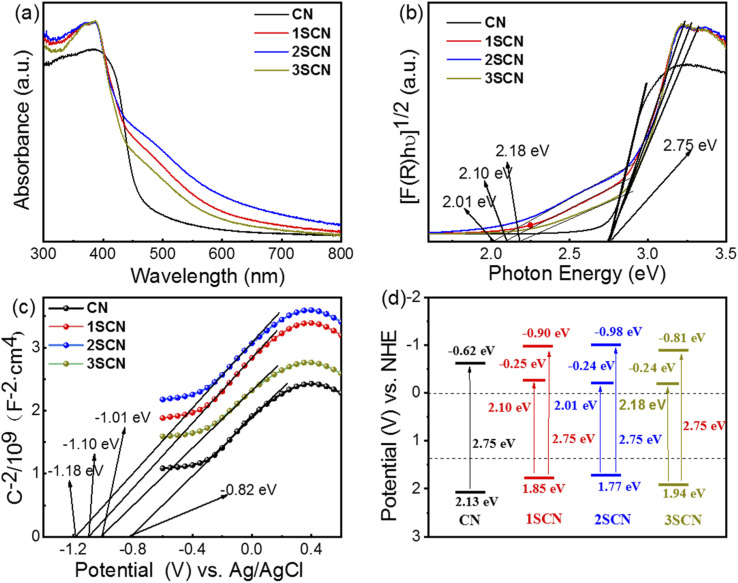
UV-vis diffuse reflectance spectra (a), converted Kubelka–Munk plots (b), Mott–Schottky plots (c), and Schematic band gap structures (d) of the samples.

As shown in Fig. 5(a), light harvest intensity at visible light range follows the sequence: 2SCN > 1SCN > 3SCN > CN. Samples with nitrogen defects exhibit strong absorption and extended visible light absorption with wide shoulder tail, denoted as Urbach tail.^39,40^ The Urbach tail is attributed to the electronic states located within the band gap, known as midgap states.^18^ According to the Kubelka–Munk function plot shown in Fig. 5(b), the intrinsic band gap value is 2.75 eV for all samples. That means that introduction of N defects increased the visible light absorption, with the intrinsic electronic structure less affected. Mott–Schottky plots in Fig. 5(c) showed that the slopes of the fitted curves of all samples are positive, indicating that the samples are n-type semiconductors.^40^ The flat band potential *vs.* Ag/AgCl is −0.82 eV, −1.01 eV, −1.10 eV and −1.18 eV, for CN, 1SCN, 2SCN, and 3SCN, respectively. Then the VB (valence band) and CB (conduction band) position is calculated by adding CB potential with band gap. The midgap is 2.18 eV, 2.10 eV, and 2.01 eV, for 3SCN, 1SCN, and 2SCN, respectively, by the Kubelk-Munk method in Fig. 5(b). The energy band structure of all samples was illustrated in Fig. 5(d). As the midgap states are close to the edge of CB, the electrons can be more easily excited from VB to midgap states. Nitrogen defects in g-C_3_N_4_ induced the formation of deeper midgap states to accommodate more charge carriers excited by photons of longer wavelengths, consistent with UV-vis results.

**Fig. 6:**
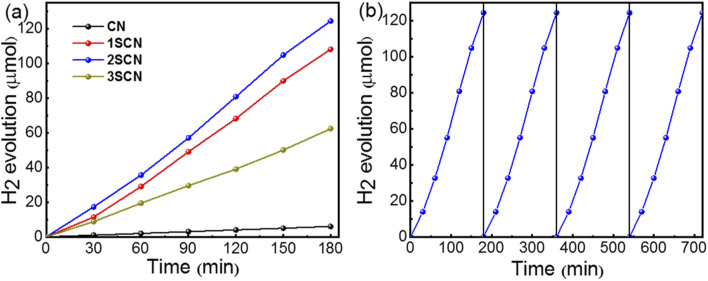
Hydrogen evolution (a) of the samples under visible light irradiation. (b) Stability of SCN600.

Fig. 6(a) shows the hydrogen evolution rate of pure g-C_3_N_4_ is 2.0 μmol h^−1^ under visible light irradiation (*λ* ≥ 420 nm). While the hydrogen evolution rate of 2SCN reached 41.4 μmol h^−1^, about 20.7 times of that of pure g-C_3_N_4_. The average quantum efficiency of 2SCN reaches 11.1%, while the average quantum efficiency of pure g-C_3_N_4_ is only 0.3%. It demonstrates that the introduction of nitrogen defects can greatly improve the photocatalytic performance of the catalysts. In order to verify the stability of the sample, the 2SCN was cycled under the same experimental conditions as shown in Fig. 6(b). After four cycles, the hydrogen evolution performance of the sample did not change, which proved that the photocatalysis of the sample was stable and could be reused.

**Fig. 7:**
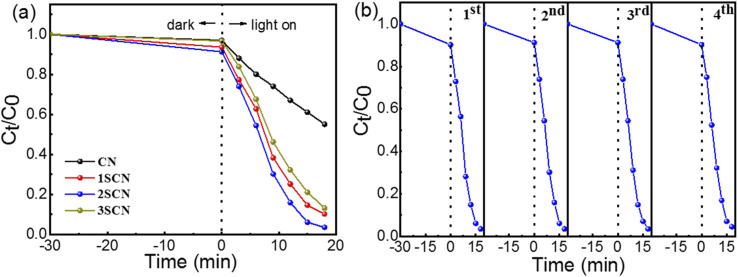
Photocatalytic degradation of RhB (a) under visible light irradiation. (b) Stability of 2SCN.

Fig. 7 (a) showed the photocatalytic degradation of RhB of the catalysts under visible light irradiation (*λ* ≥ 420 nm). The photocatalytic degradation rate reaches 45%, 90%, 97%, and 87% in 18 minutes, for CN, 1SCN, 2SCN, and 3SCN, respectively. The degradation rate of 2SCN is about 2.5 times of that of CN. In order to verify the stability of its degradation activity, the catalyst of 2SCN was tested repeatedly for four times. Our results were comparable to that of MoS_2_.^41^ As shown in Fig. 7(b), the photocatalytic activity of the sample was still stable after four cycles of testing, indicating that the catalyst had stable physical and chemical properties.

**Fig. 8:**
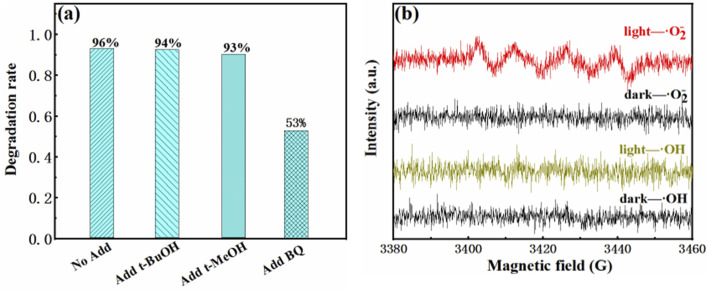
Degradation rate of 2SCN in the presence of various scavengers (a). EPR spectra in a methanol dispersion for DMPO-˙O_2_^−^ and in an aqueous dispersion for DMPO-˙OH (b).

The degradation rate did not change obviously by adding *tert*-butyl alcohol (*t*-BuOH) and methanol (MeOH), as shown in Fig. 8(a), which indicated hydroxyl radicals and holes are not the reactants for the degradation of RhB. While the photocatalytic reactions were inhibited dramatically by adding benzoquinone (BQ), indicating the radical superoxide (˙O_2_^−^) is the active radicals in the RhB degradation process. DMPO (5,5-dimethyl-1-pyrrole N-oxide) was used as a free radical trapping agent in electron paramagnetic resonance (EPR) analysis to detect free radical intermediates (˙OH and ˙O_2_^−^) generated under specific potential. Upon visible light irradiation, 2SCN in the methanol dispersion system showed obvious 1 : 1 : 1 : 1 peaks corresponding to ˙O_2_^−^ and no signals corresponding to ˙OH were observed, as shown in Fig. 8(b). The experimental results proved that the photoinduced electron–hole pairs react with adsorbed O_2_ to form ˙O_2_^−^.^42^

**Fig. 9:**
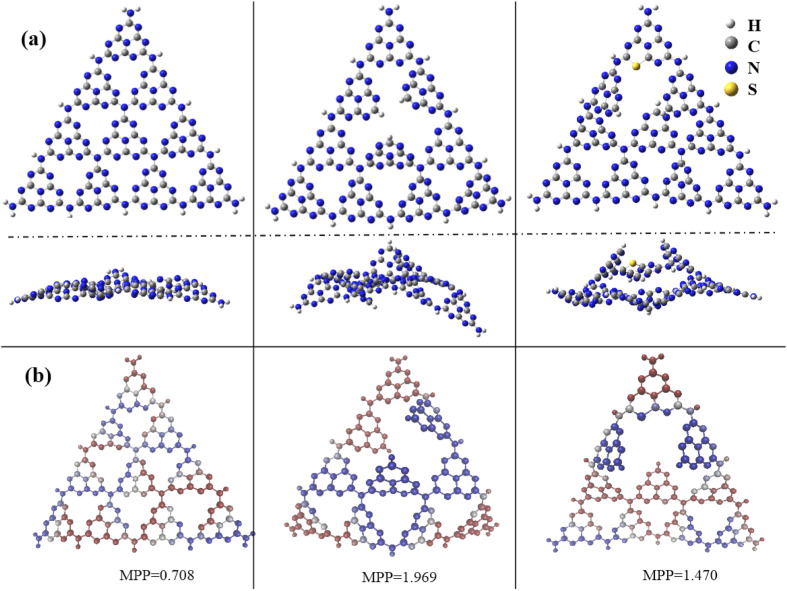
Front and side view of optimized structures of pure (left), N-defected (middle), and SCN (right); (b) quantitative measurement of planarity of three molecules. The molecular flatness parameter (MPP) is the root mean square deviation of the individual atomic distances from the fitted plane. The structure (iso-value of 0.001 a.u.) obtained at the B3LYP/6-31G* level.

Experimental results verified that nitrogen defects were introduced in g-C_3_N_4_, and a trace amount of S atoms were doped as well, which improved the transfer and separation of photoinduced carriers and photocatalytic activity. Further theoretical calculations were performed to understand the effects of N defects and S doping on the electronic structure and catalytic performance of g-C_3_N_4_. The pure g-C_3_N_4_, N-defected g-C_3_N_4_, and SCN were built up by using GaussView Rev 5.0.8,^43^ as shown in Fig. 9(a). Gaussian 09 Rev E.01 software package^44^ were selected to perform structural optimization and obtain frontier molecular orbitals including the highest occupied molecular orbital and lowest unoccupied molecular orbital (HOMO/LUMO). All calculations were at the B3LYP/6-31G* theoretical level.^45,46^ Meanwhile, we used the Multiwfn Rev 3.7 software package^45^ to gain molecular planarity, the van der Waals volume and surface area. Obviously, as shown in Fig. 9(b), the N defects and S doping make the planarity of the structures worse, increasing the molecular flatness parameter (MPP) by introducing N defects. Furthermore, to explain the performance of catalysis of these compounds, the van der Waals volume and surface area, and molecule specific surface area (SSA) were calculated and presented in Table 2 and Fig. S1.† The order of molecular volume and surface area is pure g-C_3_N_4_ < N-defected g-C_3_N_4_ < SCN. However, due to the smaller molecular mass, the order of SSA of these compounds is pure g-C_3_N_4_ < SCN < N-defected g-C_3_N_4_. Not surprisingly, all these compounds have very large SSAs (>4100 m^2^ g^−1^), which are larger than that of pure g-C_3_N_4_.

**Table 2:** The molecule volume, surface area, and molecular specific surface area of the three molecules

	g-C_3_N_4_	N-defected g-C_3_N_4_	SCN
Molecule volume (Å^3^)	1678.03	1705.80	1719.66
Molecule surface area (Å^2^)	1314.10	1393.87	1400.70
Specific surface area (m^2^ g^−1^)	4109.55	4384.06	4364.50

Next, Fig. S2† demonstrates the maps of density of states of all three compounds. Clearly, the valence bands of both pure g-C_3_N_4_, N-defected g-C_3_N_4_, and SCN are mainly composed of atomic orbitals of N atoms. And the conduction bands of both pure g-C_3_N_4_ and N-defected g-C_3_N_4_ are contributed by atomic orbitals of C + N atoms, and that of S-doped g-C_3_N_4_ with N defection consists of the atomic orbitals of C + N + S atoms. Remarkably, the HOMO energy of SCN is much higher than both of other two compounds.

**Fig. 10:**
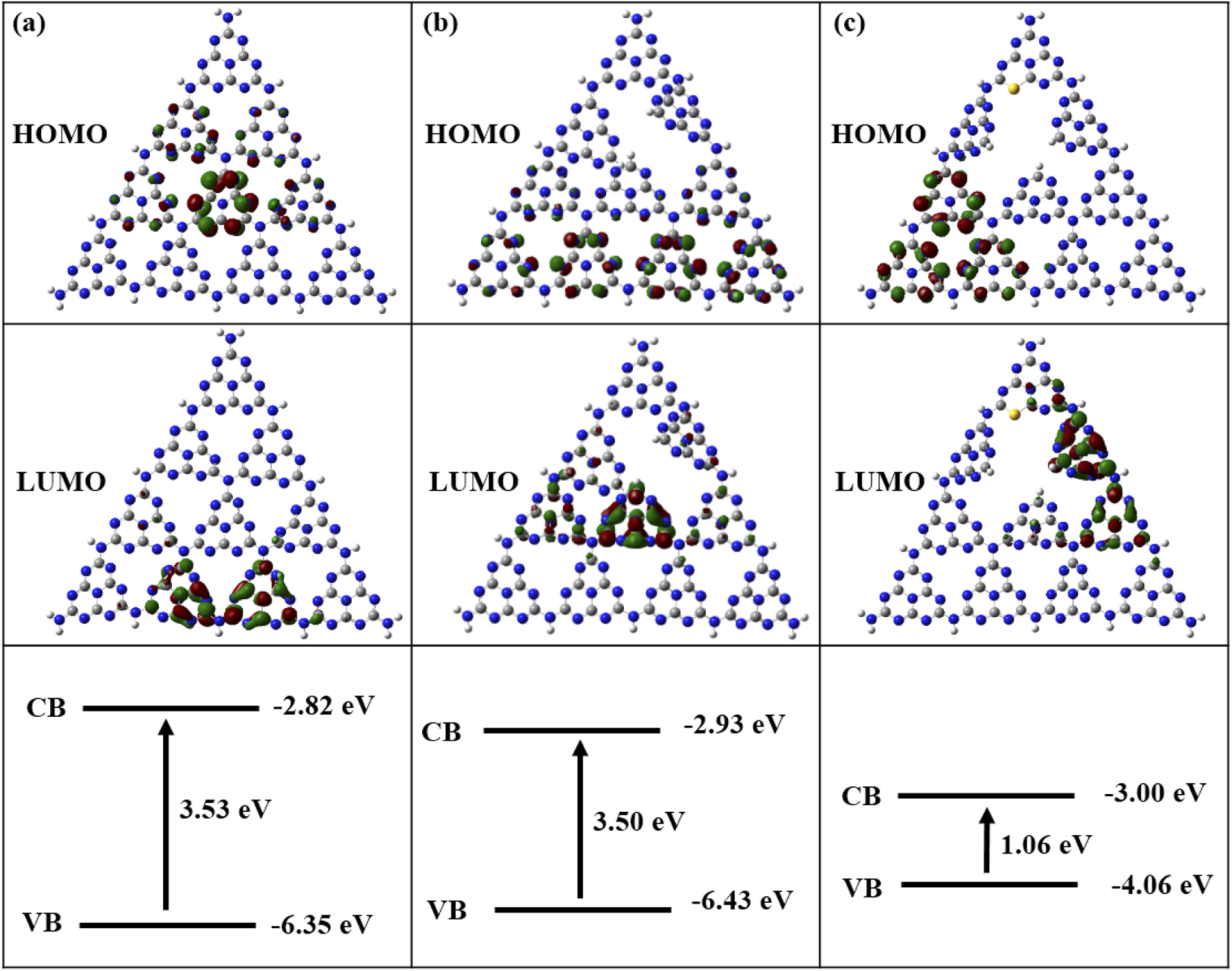
HOMO, LUMO, and gap energy of pure (a), N defected (b), and SCN (c) obtained at B3LYP/6-31G* theory level (iso-value of 0.03 a.u.). The color of codes: C atom-gray, N atom-blue, and S atom-yellow.

The HOMO and LUMO images and energy gaps of three compounds are given in Fig. 10. In Fig. 10(a), the HOMO (holes after photoexcitation) map of pure g-C_3_N_4_ indicates that the central C and N atoms provide oxygen oxidation sites, while the LUMO (excited electrons after photoexcitation) shows that the edge C and N atoms are the reduction sites for H_2_. Fig. 10(b) displays that the N defections in g-C_3_N_4_ changes the positions of oxidation and reduction sites, which are opposite to those of pure g-C_3_N_4_. Fig. 10(c) shows that the combination of N defections and S introductions cause in a clear separation of HOMO and LUMO. Compared to pure g-C_3_N_4_, its carrier mobility becomes faster with the longer separation of HOMO (holes) and LUMO (electrons). The band gaps of pure, N-defective, and SCN are 3.53 eV, 3.50 eV, and 1.06 eV, respectively. The band gap of N-defective g-C_3_N_4_ is slightly reduced compared to that of pure g-C_3_N_4_, and the electron potential well caused by N defects is conducive to electron capture and inhibits electron–hole recombination.

## 4. Conclusions

In summary, graphite carbon nitride with certain amount of nitrogen defects was synthesize by calcining melamine and thiocyanate acid at different temperatures. For SCN, the percentage of N–[C]_3_ decreases and the percentage of –NH_2_ increases with increase the ratio of thiocyanate acid, resulting in the formation of nitrogen defects in inner S–[C]_3_ linkage. The nitrogen defects induced the midgap states, which provides capture sites for photogenerated carriers, thus effectively preventing the recombination of photogenerated electrons and holes. Therefore, the quantum efficiency of the photocatalyst with nitrogen defect has been greatly improved. DFT modulations verified that N-defective g-C_3_N_4_ has a slightly reduced band gap compared to that of pure g-C_3_N_4_, and deteriorated molecular flatness and increased specific surface area caused by N defects is conducive to electron capture and inhibits electron–hole recombination, thus enhanced the photocatalytic activity of g-C_3_N_4_.

## Author contributions

The manuscript was written through contributions of all authors. All authors have given approval to the final version of the manuscript.

## Conflicts of interest

There is no conflicts to declare.

## Supplementary Material

RA-012-D2RA04928G-s001
