# Supplementary material for: Nitrogen defect-containing polymeric carbon nitride for efficient photocatalytic H2 evolution and RhB degradation under visible light irradiation
Source: RSC Adv. 2022 Aug 31;12(38):24713–23. doi: 10.1039/d2ra04928g (PMC9428898; doi:10.1039/d2ra04928g)
Supplement: RA-012-D2RA04928G-s001 [file RA-012-D2RA04928G-s001.pdf]

Electronic Supplementary Material (ESI) for RSC Advances.
This journal is © The Royal Society of Chemistry 2022

Nitrogen defected polymeric carbon nitride for efficient photocatalytic H_2 evolution and RhB degradation under visible light irradiation

Man Li‡, Xin Bai‡, Xi Rao, Shaohui Zheng, and Yongping Zhang**

School of Materials and Energy, Southwest University, Chongqing 400715, China

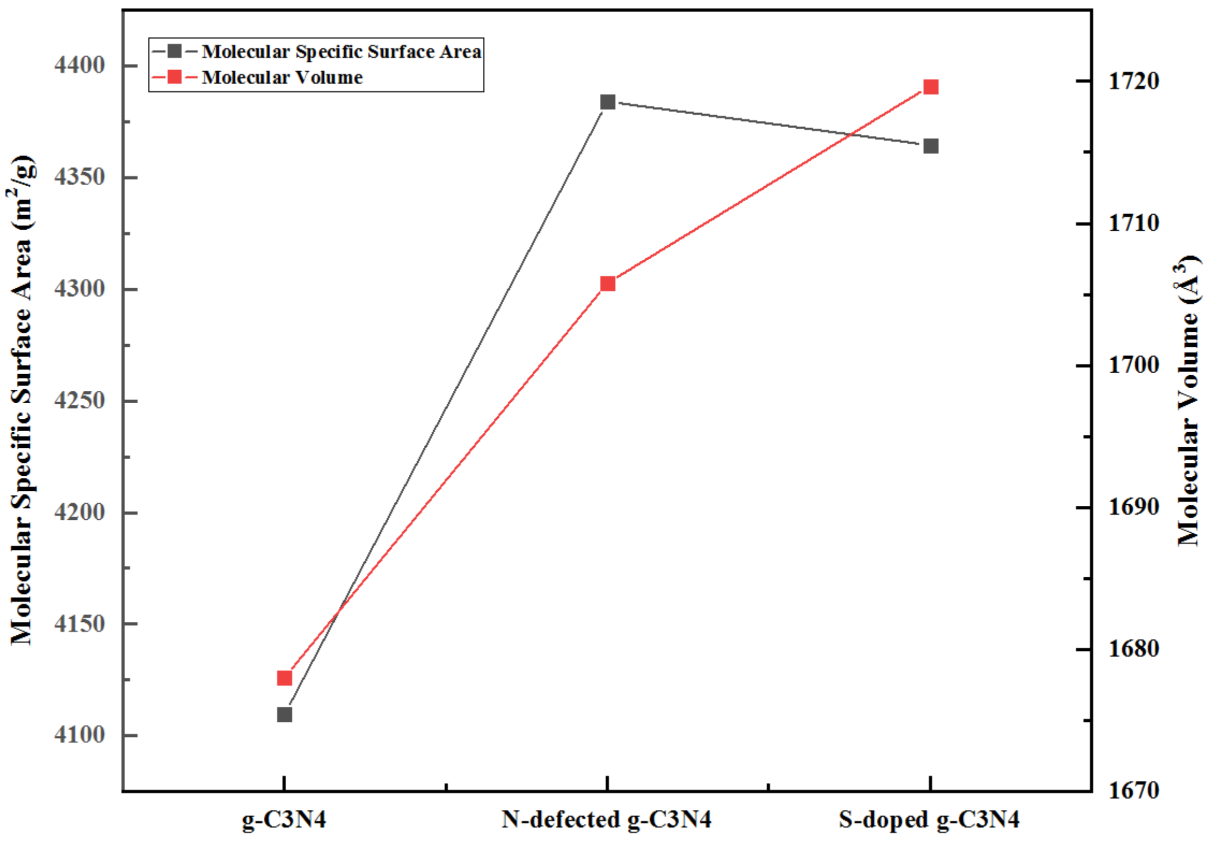

Figure S1. Calculated specific surface areas and molecular volumes of the three molecules.

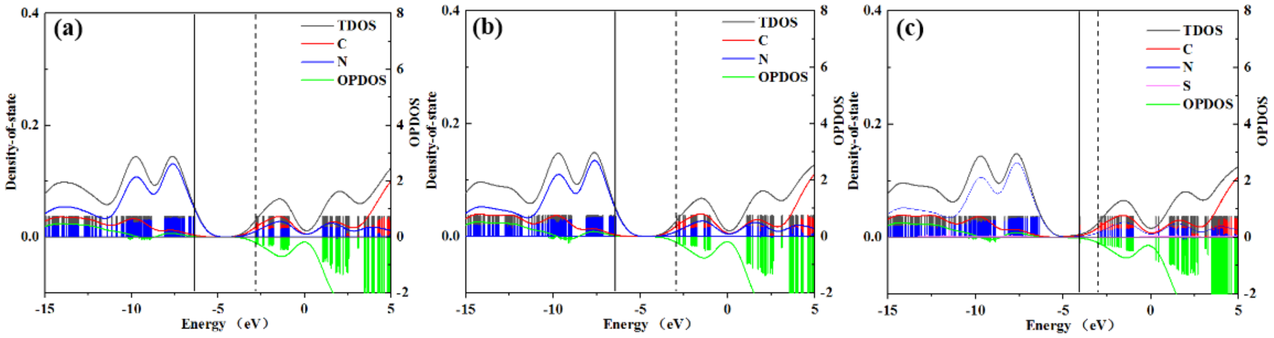

Figure S2. Calculated total, partial, and overlapped population density of states of (a) pure, (b) N-defected, and (c) S-doped g-C_3N_4 with N defection at the B3LYP/6-31G* theory level. Solid line: HOMO energy; dashed line: LUMO energy.

Table.S1 Comparison of the reported hydrogen evolution rate (HER) of g-C_3N_4 photocatalysts from different literatures.

Photocatalyst (precursors)	HER rate [μmol/(h▪g)]	Light source	Reaction conditions	Reference
2SCN (melamine and trithiocyanuric acid)	4140	500 W Xe lamp, λ > 420 nm	3 wt% Pt 17 vol% TEOA	This work
g-C_3N_4 microwire (melamine)	1688	500 W Xe lamp, λ > 380 nm	1 wt% Pt 17 vol% TEOA	[5]
g-C_3N_4 (urea)	3327	300 W Xe lamp, λ >420 nm	3 wt% Pt TEOA	[4]
g-C_3N_4 (melamine and urea)	3100	300 W Xe lamp, λ ≥ 400 nm	3 wt% of Pt 20 vol% TEOA	[2]
g-C_3N_4 (dicyandiamide)	310	300 W Xe lamp, λ > 440 nm	3 wt% Pt 10 vol% TEOA	[1]
g-C_3N_{4-x} (melamine)	3068	500 W Xe lamp, λ > 420 nm	2 wt% Pt 10 vol% TEOA	[7]
g-C_3N_4 (melamine)	1288	300 W Xe lamp, λ > 420 nm	3 wt% Pt 15 vol% TEOA	[6]
P/g-C_3N_4 (melamine)	1596	300 W Xe lamp, λ ≥ 400 nm	2 wt% Pt 20 vol% TEOA	[3]

References

[1] Niu, P.; Qiao, M.; Li, Y.; Huang, L.; Zhai, T. Distinctive defects engineering in graphitic carbon

nitride for greatly extended visible light photocatalytic hydrogen evolution. Nano Energy **2018**, 44, 73-81.

[2] Ruan, D.; Kim, S.; Fujitsuka, M.; Majima, T. Dfects rich g-C_3N_4 with mesoporous structure for efficient photocatalytic H_2 production under visible light irradiation. Appl. Catal. B **2018**, 238, 638-646.

[3] Ran, J.; Ma, T.Y.; Gao, G.; Du, X.; Qiao, S. Z. Porous P-doped graphitic carbon nitride nanosheets for synergistically enhanced visible light photocatalytic H_2 production. Energy Environ. Sci. **2015**, 8, 3708-3717.

[4] Martin, D.J.; Qiu, K.; Shevlin, S.A.; Handoko, A.D.; Chen, X.; Guo, Z.; Tang, J. Highly efficient photocatalytic H_2 evolution from water using visible light and structure-controoled graphitic carbon nitride. Angew. Chem. **2014**, 126, 9394-9399.

[5] Dou, H.; Long, D.; Zheng, S.; Zhang, Y. A facile approach to synthesize graphitic carbon nitride microwires for enhanced photocatalytic H_2 evolution from water splitting under full silar spectrum. Catal. Sci. Technol. **2018**, 8, 3599-3609.

[6] Tu, W.; Xu, Y.; Wang, J.; Zhang, B.; Zhou, T.; Yin, S.; Wu, S.; Li, C.; Huang, Y.; Zhou, Y.; Zou,Z.; Roberson, J.; Kraft, M.; Xu, R. Investgating the role of tunable nitrogen vacancies in graphitic carbon nitride nanosheets for efficient visible-light-driven H_2 evolution and CO_2 reduction. ACS Sustainable Chem. Eng. **2017**, 5, 7260-7268.

[7] Zhang, Y.; Gao, J.; Chen, Z. A solid-state chemical reduction approach to synthesize graphitic carbon nitride with tunable nitrogen defects for efficient visible-light photocatalytic hydrogen evolution, J. Colloid Interface Sci. **2019**, 535, 331-340.
